# Upfront Surgery versus Neoadjuvant Therapy for Resectable Pancreatic Cancer: Systematic Review and Bayesian Network Meta-analysis

**DOI:** 10.1038/s41598-019-40951-6

**Published:** 2019-03-13

**Authors:** Alison Bradley, Robert Van Der Meer

**Affiliations:** 1 University of Strathclyde, Department of Management Science, Glasgow, G4 0QU Scotland United Kingdom; 2 West of Scotland Pancreatic Cancer Unit, Glasgow Royal Infirmary, Glasgow, G4 0SF Scotland United Kingdom

## Abstract

Current treatment recommendations for resectable pancreatic cancer support upfront resection and adjuvant therapy. Randomized controlled trials offering comparison with the emerging neoadjuvant approach are lacking. This review aims to compare both treatment strategies for resectable pancreatic cancer. PubMed, MEDLINE, Embase, Cochrane Database and Cochrane Databases were searched for studies comparing neoadjuvant and surgery-first with adjuvant therapy for resectable pancreatic cancer. A Bayesian network meta-analysis was conducted using the Markov chain Monte Carlo method. Cochrane Collaboration’s risk of bias, ROBINS-I and GRADE tools were used to assess quality and risk of bias of included trials. 9 studies compared neoadjuvant therapy and surgery-first with adjuvant therapy (n = 22,285). Aggregate rate (AR) of R0 resection for neoadjuvant therapy was 0.8008 (0.3636–0.9144) *versus* 0.7515 (0.2026–0.8611) odds ratio (O.R.) 1.27 (95% CI 0.60–1.96). 1-year survival AR for neoadjuvant therapy was 0.7969 (0.6061–0.9500) *versus* 0.7481 (0.4848–0.8500) O.R. 1.38 (95% CI 0.69–2.96). 2-year survival AR for neoadjuvant therapy was 0.5178 (0.3000–0.5970) *versus* 0.5131 (0.2727–0.5346) O.R. 1.26 (95% CI 0.94–1.74). 5-year AR survival for neoadjuvant therapy was 0.2069 (0.0323–0.3300) *versus* 0.1783 (0.0606–0.2300) O.R. 1.19 (95% CI 0.65–1.73). In conclusion neoadjuvant therapy may offer benefit over surgery-first and adjuvant therapy. However, further randomized controlled trials are needed.

## Introduction

Pancreatic cancer (PC) is the fourth and fifth most common cause of cancer deaths in the USA and Europe respectively^1,2^. Despite advances in surgical technique and adjuvant treatment, survival rates remain poor^1,2^. Early complete surgical resection is the only potentially curative treatment for PC and adjuvant therapy has been proven to prolong survival leading to surgery first with adjuvant therapy (SFadj) becoming the standard of care for resectable pancreatic cancer (RPC)^3^. However in reality most patients develop early recurrence, nullifying the potential benefits of high-risk surgery^4^ with up to 50% of patients failing to receive adjuvant therapy due to: post-operative complications, early metastases, reduced performance status and comorbidities^5^. This has resulted in the advent of neoadjuvant therapy (NAT) with the postulated benefits of: identifying aggressive tumour hence avoiding futile surgery, elimination of micrometastesis, increased feasibility of R0 resection and completion of multimodal treatment^6,7^.

NAT for RPC is an area of prime controversy and ongoing debate with a lack of large prospective randomised controlled trials (RCTs) offering direct comparison with SFadj approach^8^. Ambiguity surrounding the existing body of research has led critics to highlight the limitations of drawing optimistic conclusions from small studies that are underpowered and caution against loosing the window of resectability^6,7^. Although not their sole focus, meta-analysis by both Xu *et al*.^9^ and Andriulli *et al*.^10^ report only marginal benefit of NAT in terms of overall and disease-free survival in RPC^7^, whilst other studies report superiority of NAT approach for RPC^11–14^. Previous Markov decision analysis studies have reported slight benefit with NAT^11,13,15^. Often comparison studies include borderline resectable and locally advanced PC in NAT arm hence they do not offer a true like-for-like comparison.

In the clinical setting the role of NAT has widely been accepted for the management of locally advanced and borderline resectable cases of PC to increase the likelihood of achieving resection, particularly R0 resection^6–8,13^. However, ambiguities in the existing body of research concerning the management of RPC with either SFadj or NAT approach creates a dilemma in clinical decision-making. It has been established that optimal survival outcomes for PC are not obtained by resection alone, but require the delivery of additional treatment whether delivered as neoadjuvant or adjuvant therapy^3,9–15^. Both SFadj and NAT treatment approaches carry the risk of failing to achieve multimodal treatment delivery. The currently recommended standard of care for RPC, SFadj^3^, carries the risk of failing to receive adjuvant therapy despite having undergone surgery with its associated risks of morbidity and mortality^4,5^. NAT approach also carries the risk of disease that was initially resectable at presentation progressing to become unresectable which makes its role in the management of RPC controversial^6,7^. The question therefore arises as to whether NAT represents a less superior treatment approach to SFadj for RPC, or if NAT has the advantage of identifying aggressive tumour types, that would have resulted in early disease reoccurrence precluding adjuvant therapy, being identified prior to patients undergoing high-risk, costly yet futile surgery^6,7^. The aim of this review is to compare SFadj and NAT approach to the management of RPC on an intention-to-treat basis. Treatment outcomes include: R0 resection rates and 1, 2, 3, 4 and 5-year survival.

## Methods

The protocol for this review was published in the PROSPERO online database of systematic reviews (CRD42018108673). This review followed the PRISMA checklist^16^.

A search was undertaken using MEDLINE, Embase, PubMed and Cochrane database. For each of the four searches, the entire database was included since 2000 up to and including 31^st^ August 2018, with no further date restrictions or limits applied. Full search strategies and are detailed in supplementary material (Supplementary Methods1).

### Search Strategy

After removal of duplicates, manual screening was carried out based on the title and abstract of articles identified in the database searches. Articles of probable or possible relevance to this review based on the title and abstract were reviewed in full. Following screening, reference lists and citations of all included papers were manually searched to identify any additional articles. This process was repeated until no new articles were identified.

#### Inclusion Criteria and Outcomes

RCTs and Prospective phase II and III trials offering comparison of NAT and SFadj approach for RPC, published in English language since 2000, involving chemo/radiotherapy-naive human subjects over 18 years of age with preoperatively staged RPC, or that reported outcomes for RPC separately, were included. As this produced only 2 studies prospective and retrospective cohort studies comparing NAT and SFadj, with the same inclusion criteria, were also included. RCTs comparing SFadj and surgery alone, with similar inclusion criteria, were also included for sensitivity analysis. Included trials had to report: protocol design, treatment regimes, number per arm, median age and co-morbidities of subjects, pre-treatment stage of pancreatic cancer, outcome from post NAT re-staging, surgical outcomes including resection rates, R0 resection rates and survival time. Case series and case reports were excluded, as were studies from identical patient cohorts. Studies that included borderline resectable, locally advanced and stage IV pancreatic cancer where results were inseparable, trials involving intra-operative radiotherapy and trials including disease other than pancreatic cancer were excluded.

### Data collection

Search design and data extraction were performed by the lead reviewer and with second author performing independent quality assurance. Discrepancies were resolved by discussion between the reviewers. The following data was extracted from each study: study details (country, year, design, number of participants, mean age, sex, co-morbidity profile and presenting disease stage of participants in each arm), details of treatment protocols, treatment outcomes (rates of tumour resection, R0 resection rates, overall survival and disease free survival and 1, 2, 3, 4 and 5 year survival rates) and risk of bias data.

The Cochrane Collaboration’s risk of bias tool^17^ and ROBINS-I tool (Risk Of Bias In Non-randomized Studies - of Interventions)^18^. Grading of Recommendations Assessment Development and Evaluation (GRADE) tool was used to provide additional assessment of quality of evidence and rate certainty in estimates from network meta-analysis^19,20^.

### Statistical analysis

This study was conducted on an intention-to-treat basis. Patients who dropped out, or who failed to receive multimodal treatment within, either SFadj or NAT pathways in the included studies were included in the overall and disease free survival analysis. The number of patients in the NAT pathway who presented with RPC but failed to undergo resection, and the number of patients who underwent surgery but failed to receive adjuvant therapy, were analysed using weighted pooled estimates of proportions calculated using Freeman-Tukey arcsine square root transformation under random effects model to account for heterogeneity.

For each outcome of interest, NetMetXl was used to draw a weighted network for all treatments assessed for the specific outcomes that accounted for the study population size of each included study^21–23^. This ensured that larger studies carried a greater weight within the network. A Bayesian network meta-analysis was conducted using the Markov chain Monte Carlo method in WinBUGS 1.4.3 (MRC Biostatistics Unit, Cambridge, and Imperial College School of Medicine, London, UK). To account for the inherent heterogeneity as a result of the different chemotherapy regimes, variations in multimodal treatment completion rates and differences in reported survival outcomes, analysis was run using a random effects, in addition to a fixed effects, model using vague priors as outlined in National Institute of Clinical Excellence Evidence Synthesis Series^21,24,25^. Pairwise comparisons between interventions were also summarized to provide ranking of impact of intervention on outcome based on the surface under the cumulative ranking (SUCRA) and summarized in rankograms^21,25^.

To further minimise the impact of heterogeneity of different chemotherapy combinations, treatment completion rates and reported survival analysis on the overall analysis, convergence was assessed using the Brooks-Gelman-Rubin method and by checking whether the Monte Carlo error is less than 5% of the standard deviation of the effect estimates and between-study variance^21^. The Markov chain Monte Carlo (MCMC) Bayesian network meta-analysis was fitted with three chains as a means of checking MCMC convergence^21^. The Brooks-Gelman-Rubin method compares within-chain and between-chain variances to calculate the potential scale reduction factor with a value close to one indicating when approximate convergence is reached^21,26^.

Inconsistency assessment, the conflict between direct and indirect evidence, is crucial to any network meta-analysis^27^. In accordance with the NICE decision-support documents^28^ inconsistency was measured by comparing deviance residuals and deviance information criteria (DIC) statistic in fitted consistency and inconsistency models^21,27^. Posterior mean deviance of the individual data points in the inconsistency model were plotted against their posterior mean deviance in the consistency model to identify any loops in the treatment network where inconsistency is present^21^.

A sensitivity network meta-analysis was carried out that also included RCTs that compared SFadj and surgery only.

## Results

### Eligible studies

A total of 14224 studies were identified through search of electronic databases (Medline/PubMed: 148; Embase: 14032; Cochrane Database: 1; Cochrane Trial Registry: 43). After removal of duplicates and studies that were not relevant on review of title and abstract, 452 studies underwent full text review (Supplementary Fig. 1a). 9 studies were identified that offered comparison between NAT and SFadj for treatment of RPC^29–37^. As only 2 of these studies were phase II trials^29,30^, one of which was randomized^29^ all studies were therefore included in the network meta-analysis. 4 studies were prospective^31–34^ and 3 studies were retrospective^35–37^ (Supplementary Table 1a; Supplementary Fig. 2).

6 studies (n = 371) reported the number of cases of RPC who received NAT and progressed to surgery^29–31,33,34,37^ giving a pooled proportion of 76.08% (95% CI: 60.826–88.509). Two studies reported response to NAT^29,31^. One study reported responses for resectable cases^29^ (complete response: 0; partial response: 4/31; stable disease 8/31; disease progress 12/31; 7 unrecorded). The study by Ielop *et al*.^31^ did not report this outcome separately for resectable only cases but included borderline cases also in reporting the outcomes of response to NAT (complete response: 5/45; partial response: 13/45; stable disease 5/45). 6 studies (n = 17596) reported the number of patients in the SFadj pathway who received adjuvant therapy^31–33,35–37^ giving a pooled proportion of 63.01% (95% CI: 59.452–66.489).

For sensitivity analysis, RCTs offering comparison between surgery and adjuvant therapy versus surgery alone were also included in a separate network meta-analysis. Electronic database search identified 25332 studies (Medline/PubMed: 3165; Embase: 21810; Cochrane Database: 1; Cochrane Trial Registry: 356). 15 studies were randomized controlled trials, 5 of which offered comparison between adjuvant therapy and surgery alone and were included in the sensitivity analysis (Supplementary Fig. 1b; Supplementary Table 1b; Supplementary Fig. 2b)^38–42^.

**Figure 1:**
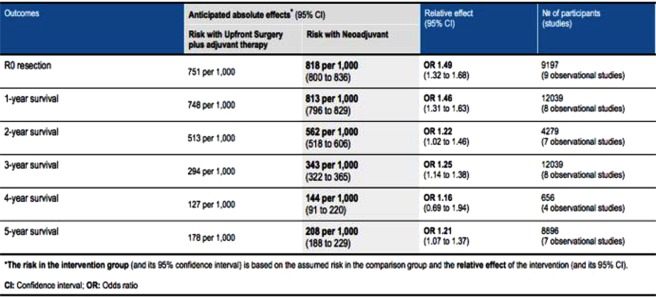
Summary of results of Bayesian network meta-analysis comparing upfront surgery and adjuvant therapy with neoadjuvant therapy for the management of resectable pancreatic cancer.

A summary of overall findings for each outcome measure is provided in Fig. 1.

### R0 Resection Rates

The network offering pairwise comparison of rates of R0 resection between NAT and SFadj included 8 studies and 9197 participant (NAT: n = 2626; SFadj: n = 6571). The aggregate rate of R0 resection for NAT was 0.8008 (0.3636–0.9144) compared to 0.7515 (0.2826–0.8611) for SFadj. Both fixed effects (O.R: 1.49; 95% CI: 1.32–1.68) and random effects (O.R: 1.27; 95% CI: 0.60–1.96) models favoured NAT. NAT was found to have superior positive impact on outcome of R0 resection (SUCRA: 0.8124 *versus* 0.1876).

### 1-year Survival Rates

Pairwise comparison for 1-year survival of NAT versus SFadj was based on 8 studies and 12011 participants (NAT: n = 2708; SFadj: n = 9303). Aggregate rate of 1-year survival was higher in NAT at 0.7969 (0.6061–0.9500) *versus* 0.7481 (0.4848–0.8500). Both fixed effects (O.R. 1.46 95% CI: 1.31–1.63) and random effects (O.R. 1.38 95% CI: 0.69–2.96) models favoured NAT. NAT also has a stronger positive impact on the outcome of 1-year survival (SUCRA: 0.84 v 0.16).

For sensitivity analysis a network also including RCTs of SFadj *versus* surgery only was constructed based on a total of 10 studies and 12483 patients (NAT: n = 2708; SFadj: n = 9540; Surgery only: n = 235). 8 studies compared NAT and SFadj (n = 12011) and 2 studies compared SFadj and surgery only (n = 472). NAT was found to be superior in both fixed and random effects models (Supplementary Fig. 3). Aggregate rate of 1-year survival was highest in NAT (0.7957; range 0.6205–0.9500) followed by SFadj (0.7478; range 0.4848–0.8500) then surgery only (0.7314; range 0.7250–0.7500). Again NAT was found to have strongest positive impact on outcome of 1-year survival (SUCRA 0.7836; Supplementary Fig. 3c).

### 2-year Survival Rates

Network pairwise comparison of NAT and SFadj for 2-year survival was based on 7 studies (n = 4251; NAT n = 903; SFadj: 3348). Aggregate rate of 2-year survival was 0.5178 (0.3000–0.5970) versus 0.5131 (0.2727–0.5346) in favour of NAT. Both fixed effects (O.R. 1.22; 95% CI: 1.02–1.46) and random effects model (O.R. 1.26; 95% CI: 0.94–1.74) favoured NAT with SUCRA 0.95 for NAT.

Inclusion of SFadj *versus* Surgery only RCTs in a network based on 9 studies (n = 4723; NAT: n = 903; SFadj: n = 3585; Surgery only: n = 235) also demonstrated superiority of NAT for 2-year survival in both fixed and random effects model (Supplementary Fig. 4). Aggregate of 2-year survival was 0.5217 (0.3000–0.5970) for NAT compared to 0.5107 (0.2727–0.5346) for SFadj and 0.4149 (0.4000–0.4200) for surgery only.

### 3-year Survival Rates

Pairwise comparison of NAT versus SFadj was based on a network comprising 8 studies (n = 12011; NAT: n = 2708; SFadj: n = 9303) and demonstrated superiority of NAT with aggregate rate of 0.3367 (0.1212–0.3900) to 0.2943 (0.1800–0.4700). Again both fixed effect (O.R. 1.25 95% CI 1.14–1.38) and random effects (O.R. 1.19 9% CI 0.86–1.51) models favored NAT with SUCRA 0.9 demonstrating stronger positive effect with NAT on outcomes of 3-year survival.

Inclusion of SFadj *versus* Surgery only RCTs in a network produced comparisons based on 9 studies (n = 12365; NAT: 2708; SFadj: n = 9482; Surgery only: n = 175). NAT was superior in both fixed and random effects models with aggregate rate 0.3400 (0.2000–0.4194) compared to 0.2951 (0.1800–0.4700) for SFadj and 0.2050 (0.2050–0.2050) for surgery only (Supplementary Fig. 5).

### 4-year Survival Rates

Only pairwise comparison of NAT and SFadj could be offered, as SFadj *versus* surgery only RCTS did not report 4-year survival rates. This network was based on 4 studies (n = 656). NAT was superior with aggregate rate 0.1416 (0.0303–0.2500) compared to 0.1269 (0.0606–0.2000). Fixed effects (O.R. 1.16 95% 0.69–1.94) and random effects model (O.R 1.03 95% CI 0.27–3.13) favored NAT.

### 5-year Survival Rates

Network pairwise comparison of 5-year survival for NAT and SFadj was based on 7 studies (n = 8896; NAT: n = 2558; SFadj: n = 6338). Aggregate rate for NAT was 0.2069 (0.0323–0.3300) compared to 0.1783 (0.0606–0.2300). Fixed effects (O.R 2.21 95% CI: 1.07–1.37) and random effects (vague prior) (O.R. 1.19 95% 0.65–1.73) favored NAT with SUCRA 0.82 for NAT association with 5-year survival.

Inclusion of SFadj *versus* surgery only RCTs was based on 11 studies (n = 9675; NAT n = 2558; SFadj n = 6730; Surgery only n = 387). NAT was superior across fixed effects and random effects models with aggregate rate 0.2069 (0.0323–0.3300) followed by 0.1814 (0.0606–0.2640) for SFadj and 0.1418 (0.1040–0.2200) for surgery only (Supplementary Fig. 6).

### Convergence, Inconsistency and Assessment of Strength of Recommendations

Convergence was achieved across all models and no issues were identified with inconsistency. In 2-year survival analysis and 5-year survival analysis there was a marginal preference towards fixed effects model as determined by the DIC statistic.

Overall this analysis marginally favors NAT for treatment of RPC across outcomes of R0 resection, 1, 2, 3, 4 and 5-year survival. This is based on the best available studies and did not alter on sensitivity analysis. However, issues pertaining to quality and level of bias of available studies are an issue that weakens the strength and level of certainty of any such recommendations (Supplementary Fig. 7).

## Discussion

SFadj is a well established treatment pathway for RPC^3^. NAT is supported by current guidelines for borderline resectable and locally advanced PC but its role in the management of RPC remains controversial^8,13^. Postulated benefits of NAT include: identifying aggressive tumour types hence avoiding futile surgery, elimination of micrometastesis, increased R0 resection rate and increased rate of completion of multimodal treatment considering that up to 50% of patients treated in SFadj pathway fail to receive adjuvant therapy^5–7^. However, controversy in the role of NAT for RPC arises from the potential of loosing the window of resectability^6,7^. In the absence of conclusive results from large multi-centered RCTs, this study, the first of its kind, utilizes existing studies comparing NAT and SFadj for the treatment of RPC in a Bayesian network meta-analysis to offer an important interim source of information to inform the ongoing debate regarding the best treatment for RPC.

In terms of survival time, from direct and indirect comparisons our analysis found that NAT was marginally superior to SFadj across 1, 2, 3, 4 and 5-year survival. These findings are corroborated by previous attempts to synthesize existing evidence comparing SFadj and NAT for RPC. Meta-analysis by both Xu *et al*.^9^ and Andriulli *et al*.^10^ reported marginal benefit of NAT for RPC in terms of OS and DFS for resectable cases. However, neither of these reports focused solely on NAT and therefore omitted significant studies from their meta-analysis^7^. Sharma *et al*.^11^ and de Gus *et al*.^13^ synthesized published data in a Markov decision-analysis model to compared NAT and SFadj for the treatment of RPC and also reported marginal benefit of NAT. More recently Versteijne *et al*.^14^ reported more significant survival benefit with NAT in their meta-analysis but the reported weighted mean overall survival time included borderline resectable cases therefore captured the effect of conversion to resectability affecting overall survival time in NAT pathway. The reported weighted mean overall survival time for resectable only cases was lower although still superior to SFadj^14^.

The second key outcome explored through direct and indirect comparison was the rate of R0 resection, which is known to impact survival time^43^. Once again NAT was found to be superior to SFadj which is in keeping with the hypothesis that NAT results in higher rates of R0 resection^6,7,44^. However, definitions of R0 resection can vary between studies, which could potentially impact reported outcomes^14^. In this study convergence was achieved across all models comparing this outcome and no issues with inconsistency were identified in our analysis.

A key clinical concern when selecting a treatment pathway for RPC is the delivery of multimodal treatment: resection in the NAT pathway and receipt of adjuvant therapy in the SFadj pathway. Our analysis of pooled proportions found that 63% of patients in the SFadj pathway received adjuvant therapy, and 76% in the NAT pathway underwent resection. These findings are in keeping with the results of a recent meta-analysis of pooled proportions that reported 68.6% of patients in SFadj received adjuvant therapy and 76.8% of resectable cases in NAT pathways underwent resection^14^.

A strength of this study is that only studies of RPC, identified through comprehensive literature search, were included to offer a true like-for-like comparison based on currently available evidence. Analysis of NAT *versus* SFadj were based on direct comparisons to strengthen certainty of findings with indirect comparisons drawn from inclusion of SFadj *versus* surgery only in sensitivity analysis which did not alter network findings. However, this study also shares the limitations of the existing body of evidence pertaining to treatment of RPC: heterogeneity and small underpowered sample size^10^. Although random effects modeling was employed to counter heterogeneity, overall there is a lack of RCTs comparing NAT and SFadj for RPC^7,10,11,13^. Only one of the two phase II trials were randomized^29^ with the remaining studies being either prospective or retrospective studies which raises serous concerns about bias and reduced certainty in the recommendations drawn from the network meta-analysis. However, unlike the majority of existing network meta-analysis^45–47^, this study went beyond only assessing bias of included trials to utilise GRADE approach to rate the certainty in estimates from our network meta-analysis^20,48–51^. Hence this study not only furthers the ongoing current debate regarding best treatment for RPC by offering an important interim analysis, but adds a further dimension by highlighting limitations of the body of evidence on which this analysis is based.

To conclude our Bayesian network meta-analysis shows that NAT for treatment of RPC is no worse than traditional SFadj approach and may even hold benefit across outcomes of: R0 resection, 1, 2, 3, 4 and 5-year survival. This finding in the context of limitations of existing studies means that conclusive superiority of one approach over another for RPC cannot be determined without a degree of uncertainty. This highlights three important directions for future research: 1) rigorous head-to-head comparison of NAT and SFadj for treatment of RPC 2) cost-effectiveness analysis of NAT *versus* SFadj and 3) exploring methods of predictive statistical modeling to identify patients who are more likely to receive and benefit from differing treatment modalities within competing pathways. By moving research in this direction it is hoped that we can find a path from ambiguity to delivering personalized medicine with associated benefits for patients and resource utilization.

## Supplementary information

Supplementary Material

## Data Availability

The datasets analysed during the current study are publicly available and sources are cited in supplementary material.
