# Supplementary material for: Upfront Surgery versus Neoadjuvant Therapy for Resectable Pancreatic Cancer: Systematic Review and Bayesian Network Meta-analysis
Source: Sci Rep. 2019 Mar 13;9:4354. doi: 10.1038/s41598-019-40951-6 (PMC6416273; doi:10.1038/s41598-019-40951-6)
Supplement: Supplementary file 1 — Supplementary Material [file 41598_2019_40951_MOESM1_ESM.pdf]

Upfront Surgery versus Neoadjuvant Therapy for Resectable Pancreatic Cancer: Systematic Review and Bayesian Network Meta-analysis

Alison Bradley[1,2*], Robert van der Meer[1]

Supplementary Methods1a. Search terms for studies comparing neoadjuvant therapy versus surgery first and adjuvant therapy.

- neoadjuvant
- treatments neoadjuvant
- treatment neoadjuvant
- therapy neoadjuvant
- therapies neoadjuvant
- neoadjuvant treatments
- neoadjuvant treatment
- neoadjuvant therapy
- neoadjuvant therapies
- induction therapy
- neoadj
- upfront
- surgery
- operative surgery
- surgery operative
- surgical procedures operative
- surgical treatment
- surgical interventions
- procedures operative surgical
- procedures operative
- procedure operative
- operative surgical procedures
- operation surgery
- operation
- surgeries
- surgery specialty
- surgical aspects
- operative therapy
- operations
- operative procedure
- surgical
- surgical procedure
- surgical procedures
- tree surgeon
- tree surgeons
- pancreatic cancer
- cancer pancreas
- cancer pancreatic
- cancers pancreas
- cancers pancreatic
- malignant neoplasm pancreas
- pancreas cancer
- pancreas cancers
- pancreatic cancers
- malignant tumor of pancreas
- malignant tumour of pancreas
- pancreatic carcinoma
- pancreas carcinoma
- exocrine pancreas carcinoma

Supplementary Methods1b: Search terms for randomized control trials: surgery only versus surgery and adjuvant therapy.

- adjuvant
- adjuvants
- pharmaceutical adjuvants
- pharmaceutical adjuvant
- adjuvant pharmaceutical
- therapy
- encounter due to therapy
- therapeutic aspects
- disease management
- treatment
- therapeutic procedure
- therapeutic interventions
- therapies
- treatments
- remedy
- relief
- amelioration
- alleviation
- remedies
- therapeutic
- relieve
- ameliorate
- alleviate
- relieving
- alleviating
- alleviated
- ameliorated
- relieved
- pancreatic cancer
- cancer pancreas
- cancer pancreatic
- cancers pancreas
- cancers pancreatic
- malignant neoplasm pancreas
- pancreas cancer
- pancreas cancers
- pancreatic cancers
- malignant tumor of pancreas
- malignant tumour of pancreas
- pancreatic carcinoma
- pancreas carcinoma
- exocrine pancreas carcinoma

Supplementary Figure1a: PRISMA flow chart: neoadjuvant therapy versus surgery first and adjuvant therapy

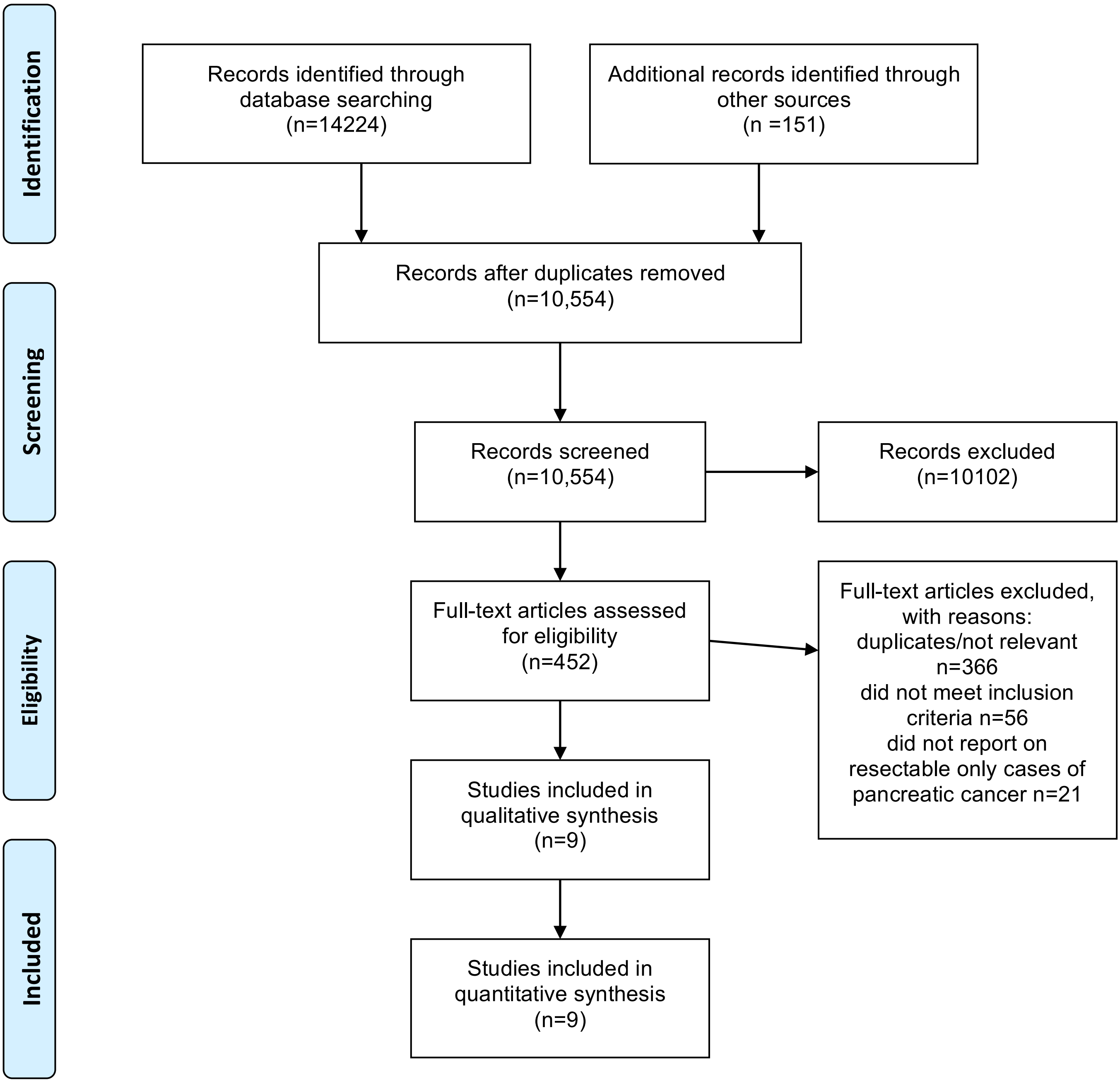

Supplementary Figure1b: PRISMA flow chart: surgery first and adjuvant therapy versus surgery only

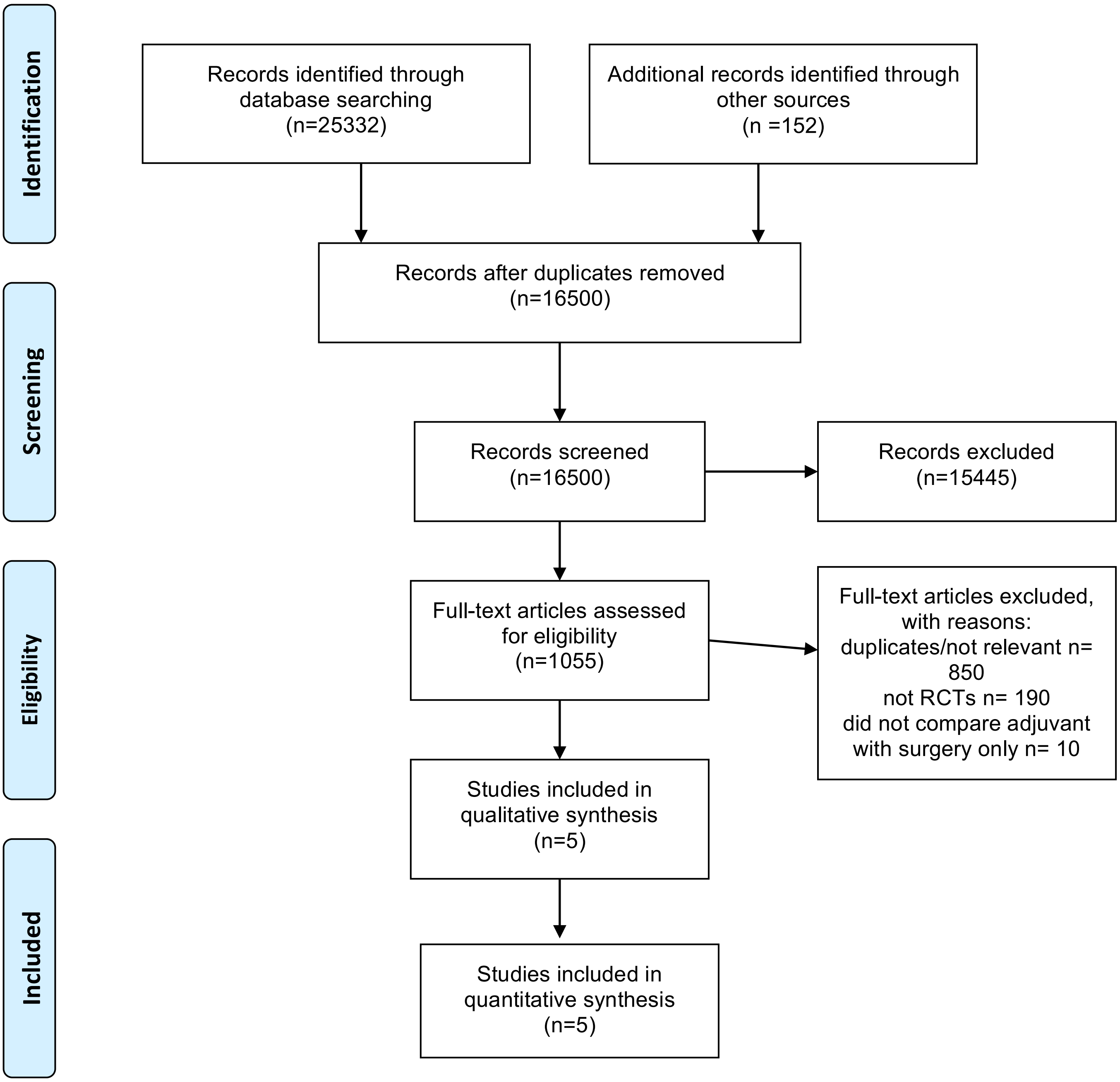

Supplementary Table1a: Summary of included studies. Summary of studies comparing neoadjuvant therapy versus surgery first and adjuvant therapy.

Study	Study Type	Randomised	Centre	NAT treatment Regime in addition to radiotherapy	Total No. patient in NAT arm	NAT arm Overall Survival in months for RPC	Total No. patients SFadj arm	SFadj arm Overall Survival in months	ROBINS-I risk of bias assessment
Golcher et al., 2015[29]	Phase II	Yes	Multiple	Gemcitabine/ cisplatin	31	17.4	33	14.4	Low
Vento et al., 2007[30]	Phase II	No	Single	Gemcitabine	22	30.2	25	35.9	Moderate
Ielpo et al., 2017[31]	Prospective	No	Single	Gemcitabine +Nabpaclitaxel	19	21.65	36	22.1	Moderate
Roland et al., 2015[32]	Prospective	No	Single	Gemcitabine, 5-FU or capecitabine	222		85		Moderate
DeGus et al., 2017[35]	Retrospective	No	Multiple (cancer registry)	NAT: no further details given	332	26	11316	24.5	Moderate /Serious
Mokdad et al., 2017[36]	Retrospective	No	Multiple (cancer registry)	NAT: no further details given	2005	26	6015	21	Moderate /Serious
Tzeng et al., 2014[33]	Prospective	No	Single	NAT: no further details given	115	28	62	25.3	Moderate /Serious
Fujii et al., 2016[34]	Prospective	No	Single	S1+5-FU+oteracil and gimeracil	40	24	416	23	Moderate /Serious
Papalezova et al., 2012[37]	Retrospective	No	Single	5-FU	144	15	92	13	Moderate /Serious

Supplementary Table1b: Summary of included studies. Summary of randomized controlled trials comparing upfront and adjuvant therapy versus surgery only.

Study	Adjuvant Regime	Adjuvant chemotherapy agents	No. SFadj arm	Overall survival in months SFadj arm	No. Surgery Only arm	Overall survival in surgery only arm
Ueno et al., 2009[38]	CT	Gemcitabine	58	22.3	60	18.4
Oettle et al., 2013[39]	CT	Gemcitabine	179	22.8	175	20.2
Kosuge et al., 2006[40]	CT	Cisplatin + 5-FU	45	12.5	44	15.8
Smeenk et al., 2007[41]	CRT	5-FU	110	21.6	108	19.2
Morak et al., 2008[42]	CRT	5-FU+folic acid+ mitoxantrone + cisplatin	59	19	61	18

Supplementary Figure2a: Assessment of risk of bias. Assessment of the risk of bias of studies comparing neoadjuvant therapy versus upfront surgery and adjuvant therapy for the treatment of resectable pancreatic cancer.

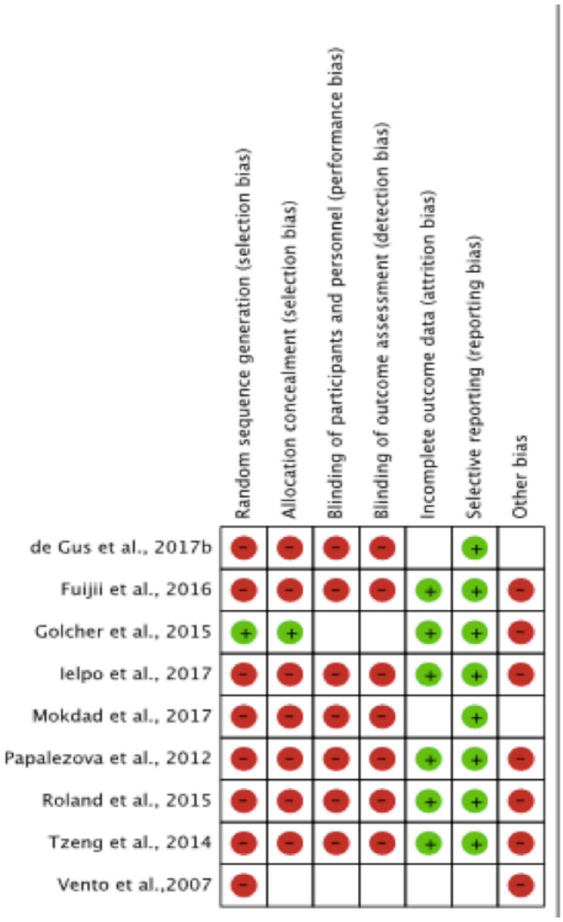

Supplementary Figure2b: Assessment of risk of bias. Assessment of the risk of bias of randomized controlled trials comparing upfront surgery and adjuvant therapy versus surgery only for resectable pancreatic cancer.

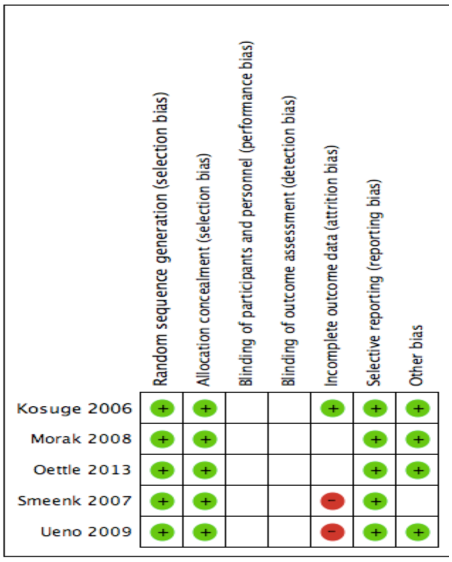

	Random sequence generation (selection bias)	Allocation concealment (selection bias)	Blinding of participants and personnel (performance bias)	Blinding of outcome assessment (detection bias)	Incomplete outcome data (attrition bias)	Selective reporting (reporting bias)	Other bias
Kosuge 2006	+	+			+	+	+
Morak 2008	+	+				+	+
Oettle 2013	+	+				+	+
Smeenk 2007	+	+			−	+	
Ueno 2009	+	+			−	+	+

Supplementary Figure3a: Results for 1-year survival. Results of fixed effects and random effects (vague prior) models.

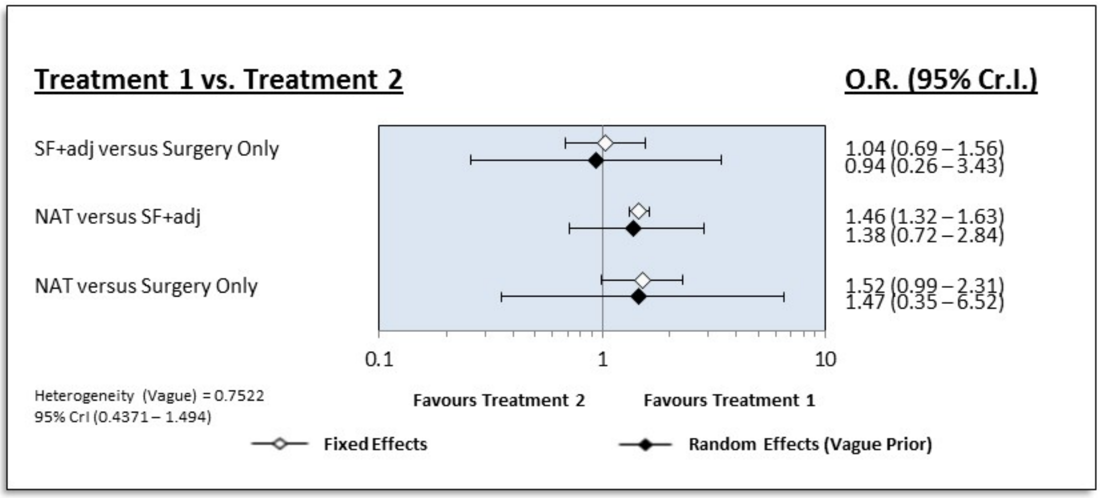

Supplementary Figure3b: Results for 1-year survival. League table based on Results of fixed effects and random effects (vague prior) models. Where the displayed odds ratio is greater than 1, treatment at top left is superior.

**NAT**		
1.46<br>(1.32 – 1.63)	**SF+adj**	
1.52<br>(0.99 – 2.31)	1.04<br>(0.69 – 1.56)	**Surgery Only**

Supplementary Figure3c: Results for 1-year survival. Rankogram summarizing SUCRA scores

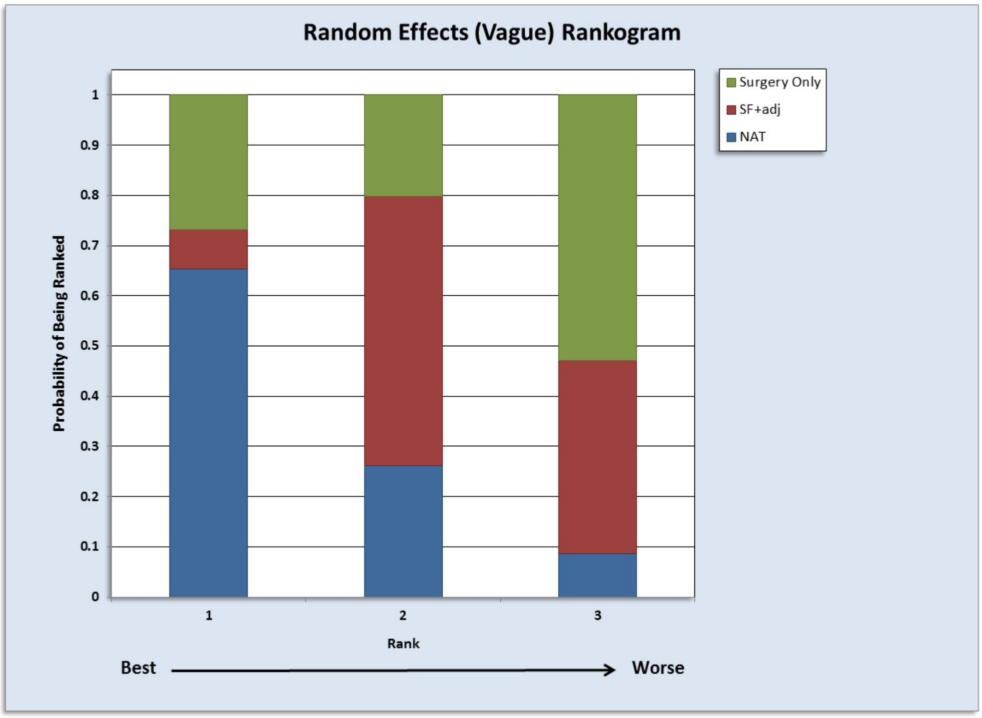

Supplementary Figure4a: Results for 2-year survival. Results of fixed effects and random effects (vague prior) models.

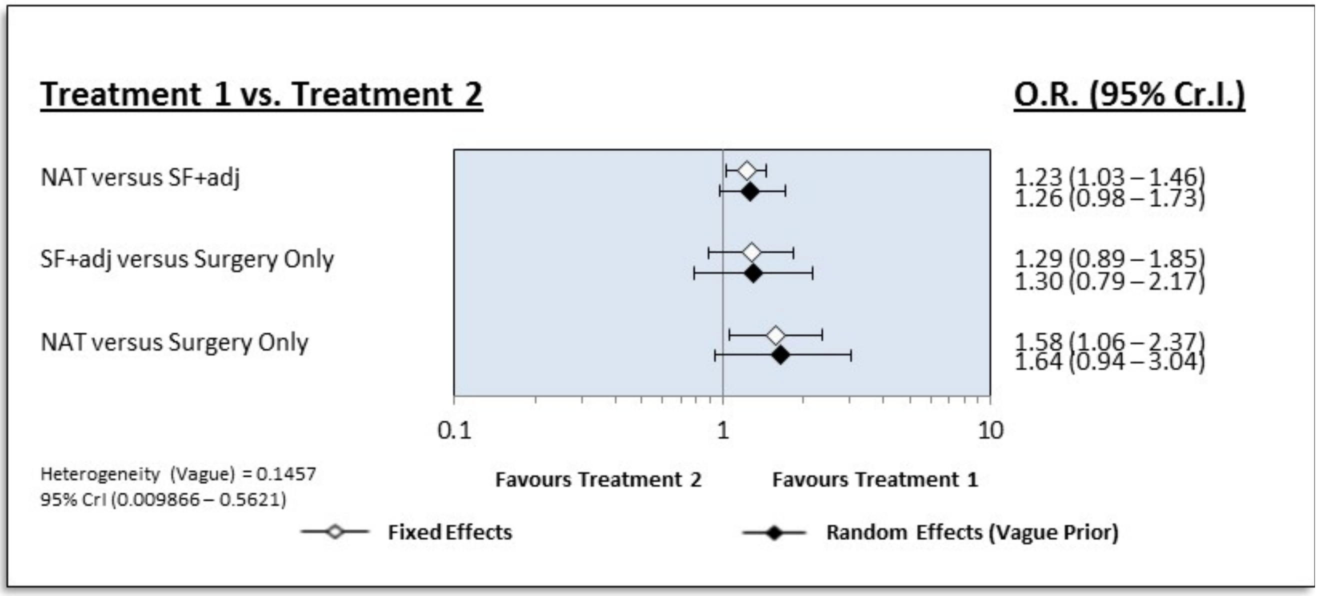

Supplementary Figure4b: Results for 4-year survival. League table based on Results of fixed effects and random effects (vague prior) models. Where the displayed odds ratio is greater than 1, treatment at top left is superior.

**NAT**		
1.23 (1.03 – 1.46)	**SF+adj**	
1.58 (1.06 – 2.37)	1.29 (0.89 – 1.85)	**Surgery Only**

Supplementary Figure4c: Results for 2-year survival. Rankogram summarizing SUCRA scores

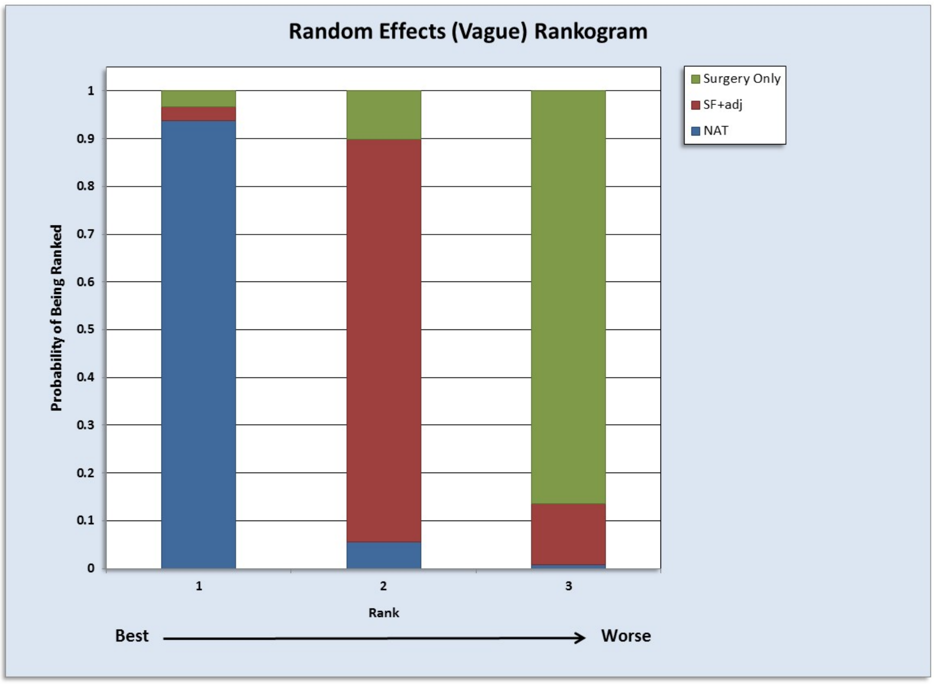

Supplementary Figure5a: Results for 3-year survival. Results of fixed effects and random effects (vague prior) models.

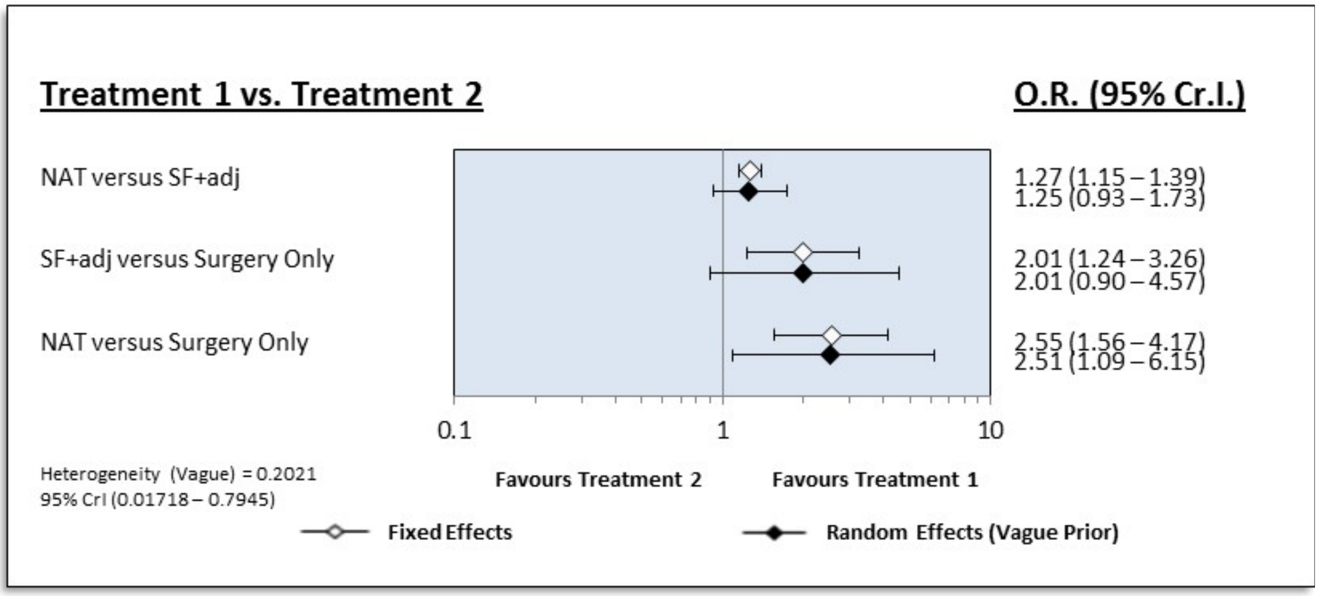

Supplementary Figure5b: Results for 3-year survival. League table based on Results of fixed effects and random effects (vague prior) models. Where the displayed odds ratio is greater than 1, treatment at top left is superior.

**NAT**		
1.27<br>(1.15 – 1.39)	**SF+adj**	
2.55<br>(1.56 – 4.17)	2.01<br>(1.24 – 3.26)	**Surgery Only**

Supplementary Figure5c: Results for 3-year survival. Rankogram summarizing SUCRA scores

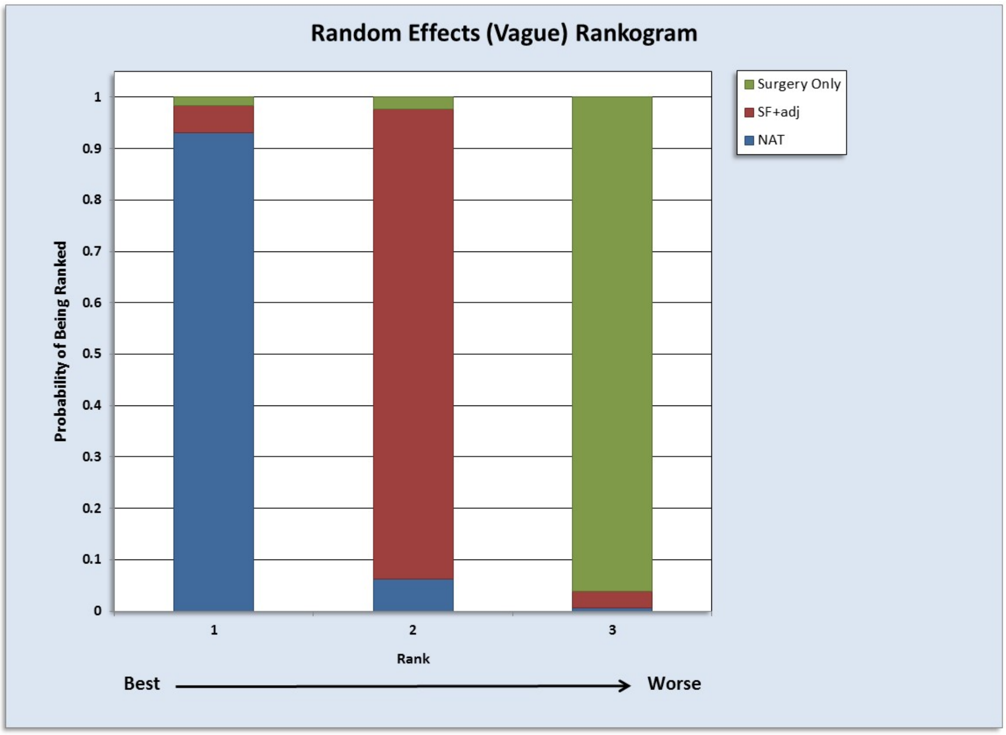

Supplementary Figure6a: Results for 5-year survival. Results of fixed effects and random effects (vague prior) models.

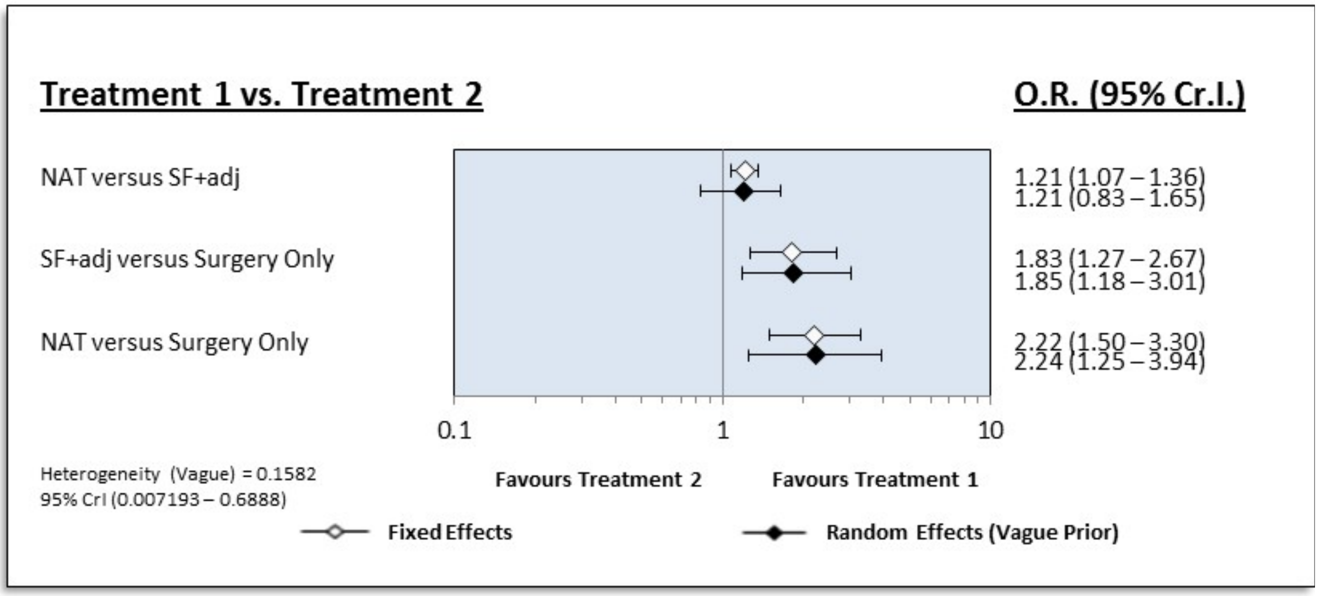

Supplementary Figure6b: Results for 5-year survival. League table based on Results of fixed effects and random effects (vague prior) models. Where the displayed odds ratio is greater than 1, treatment at top left is superior.

**NAT**		
1.21<br>(1.07 – 1.36)	**SF+adj**	
2.22<br>(1.50 – 3.30)	1.83<br>(1.27 – 2.67)	**Surgery Only**

Supplementary Figure6c: Results for 5-year survival. Rankogram summarizing SUCRA scores

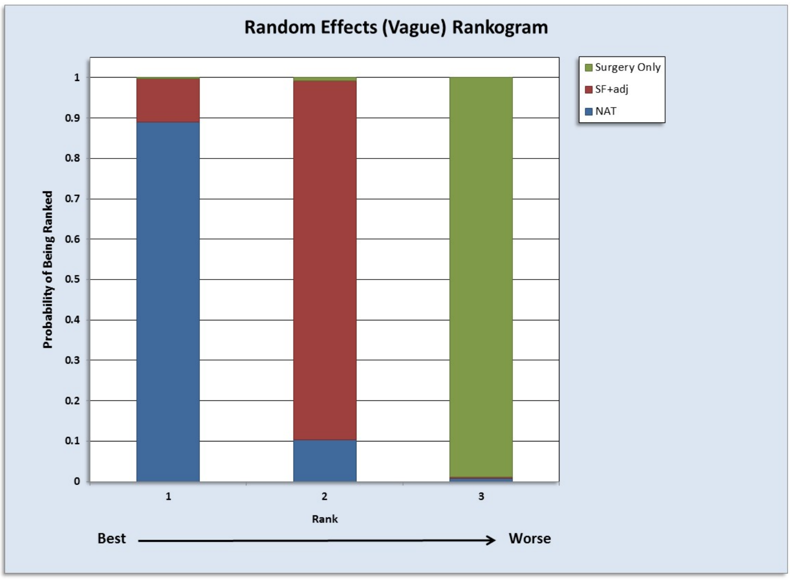

Supplementary Figure7: GRADE assessment of strength of recommendations. An assessment of the strength of overall recommendations from the network meta-analysis according to the GRADE assessment criteria.

CRITERIA	SUMMARY OF JUDGEMENTS				
PROBLEM	No	Probably no	Probably yes	**Yes**	
DESIRABLE EFFECTS	Trivial	Small	**Moderate**	Large	
UNDESIRABLE EFFECTS	Large	Moderate	**Small**	Trivial	
CERTAINTY OF EVIDENCE	Very low	Low	**Moderate**	High	
VALUES	Important uncertainty or variability	**Possibly important uncertainty or variability**	Probably no important uncertainty or variability	No important uncertainty or variability	
BALANCE OF EFFECTS	Favors the comparison	Probably favors the comparison	Does not favor either the intervention or the comparison	Probably favors the intervention	**Favors the intervention**
ACCEPTABILITY	No	Probably no	**Probably yes**	Yes	
FEASIBILITY	No	Probably no	Probably yes	**Yes**	
